# Alternative Electron Transports Participate in the Maintenance of Violaxanthin De-Epoxidase Activity of *Ulva* sp. under Low Irradiance

**DOI:** 10.1371/journal.pone.0078211

**Published:** 2013-11-08

**Authors:** Xiujun Xie, Wenhui Gu, Shan Gao, Shan Lu, Jian Li, Guanghua Pan, Guangce Wang, Songdong Shen

**Affiliations:** 1 Tianjin Key Laboratory of Marine Resources and Chemistry, College of Marine Science and Engineering, Tianjin University of Science and Technology, Tianjin, China; 2 Institute of Oceanology, Chinese Academy of Sciences, Qingdao, China; 3 Graduate School, Chinese Academy of Sciences, Beijing, China; 4 School of Life Science, Nanjing University, Nanjing, China; 5 Earth and Life Institute, Catholic University of Louvain, Louvain la Neuve, Belgium; 6 College of Life Sciences, Soochow University, Suzhou, China; University of Hyderabad, India

## Abstract

The xanthophyll cycle (Xc), which involves violaxanthin de-epoxidase (VDE) and the zeaxanthin epoxidase (ZEP), is one of the most rapid and efficient responses of plant and algae to high irradiance. High light intensity can activate VDE to convert violaxanthin (Vx) to zeaxanthin (Zx) via antheraxanthin (Ax). However, it remains unclear whether VDE remains active under low light or dark conditions when there is no significant accumulation of Ax and Zx, and if so, how the ΔpH required for activation of VDE is built. In this study, we used salicylaldoxime (SA) to inhibit ZEP activity in the intertidal macro-algae *Ulva* sp. (Ulvales, Chlorophyta) and then characterized VDE under low light and dark conditions with various metabolic inhibitors. With inhibition of ZEP by SA, VDE remained active under low light and dark conditions, as indicated by large accumulations of Ax and Zx at the expense of Vx. When PSII-mediated linear electron transport systems were completely inhibited by SA and DCMU, alternative electron transport systems (i.e., cyclic electron transport and chlororespiration) could maintain VDE activity. Furthermore, accumulations of Ax and Zx decreased significantly when SA, DCMU, or DBMIB together with an inhibitor of chlororespiration (i.e., propyl gallate (PG)) were applied to *Ulva* sp. This result suggests that chlororespiration not only participates in the build-up of the necessary ΔpH, but that it also possibly influences VDE activity indirectly by diminishing the oxygen level in the chloroplast.

## Introduction

Light is essential for photosynthesis, yet it also may be potentially harmful to plants. To prevent excess absorption of light energy and consequent oxidative damage to the photosynthetic apparatus, higher plants and most algae evolved various photoprotection mechanisms. One of the most rapid and efficient of these mechanisms is the xanthophyll cycle (Xc) [1], [2]. Its operation involves two critical enzymes: Violaxanthin de-epoxidase (VDE) catalyzes the conversion of violaxanthin (Vx) to zeaxanthin (Zx) via antheraxanthin (Ax), and Zx epoxidase (ZEP) catalyzes the reverse reaction, from Zx to Vx via Ax [3], [4], [5].

Although the enzyme VDE has been purified in higher plants and comprehensively investigated in vitro [6], [7], some questions still remain. First, it is well known that VDE and ZEP work antagonistically in vivo; thus, study of the factors that regulate the activity of VDE in vivo is often hampered by the presence of ZEP. Although ZEP mutants have been obtained in some higher plants [8], [9], [10] and green algae [11], these mutants are not very suitable for studying VDE in vivo because of deficiency of Vx in LHCII [2]. Thus, inhibiting ZEP using inhibitors in wild type organisms may be an alternative method for characterizing VDE activity in vivo. Second, although it is known that high light intensity can activate VDE to convert Vx to Zx via Ax, it is unclear whether VDE remains active under low light or dark conditions when there is no significant accumulation of Ax and Zx, and if so, how the necessary ΔpH across the thylakoid membrane for the activity of VDE is built.

*Ulva* sp. (Ulvales, Chlorophyta) is a macro-alga growing in the intertidal zone, where maximal irradiances often become higher than 1000 µmol m^−2^s^−1^. Thus, efficient photoprotection mechanisms should be crucial for its survival. Meanwhile, *Ulva* sp. is composed of two layers of cells, which may be critical for the effective infiltration of various inhibitors into the thalli. Herein, *Ulva* sp. was used to investigate the operation of the Xc under low light intensity in vivo in the presence of salicylaldoxime (SA), which inhibits the catalytic ability of ZEP. We propose that both VDE and ZEP are permanently operating in *Ulva* sp. under low light and dark conditions, and, in the latter case, the required ΔpH can be built by chlororespiration-mediated electron transport.

## Materials and Methods

### Algae samples

*Ulva* sp. (Ulvales, Chlorophyta) was collected from Zhanshan, Qingdao (36°05′N, 120°18′E), China. This location is not privately-owned or protected in any way, thus no specific permissions were required, and the field studies did not involve endangered or protected species. The thalli were rinsed with sterilized seawater to remove sand and epiphytes, and were cultured at 15°C±2°C in seawater with illumination at about 50 µmol m^−2^s^−1^ supplied with white fluorescent lamp prior to the experiments.

### Treatment with metabolic inhibitors

SA at a final concentration of 5 mM was used to inhibit the activity of ZEP [12]. This concentration does not influence the activities of plastocyanin and PSI [13]. Dithiothreitol (DTT), which inhibits VDE by reducing the disulfide bridge within VDE, was used at a final concentration of 3 mM [14]. To evaluate the effects of ΔpH on the SA-induced accumulations of Zx and Ax, carbonyl cyanide p-trifluoromethoxyphenylhydrazone (FCCP) at a final concentration of 10 µM was used to collapse the trans-thylakoid membrane proton gradient [15].

The electron transport systems along the thylakoid membrane were inhibited by 10 µM of 3-(3, 4-dichlorophenyl)-1, 1-dimethylurea (DCMU) on the PSII site [16], 10 µM of dibromothymoquinone (DBMIB) on the cytochrome b6f complex site [17], or 1 mM of propyl gallate (PG) on the plastid terminal oxidase (PTOX) site [18]. All inhibitors were prepared as 1000× stock solutions by dissolution in methanol followed by dilution with seawater. The final concentrations of organic solvents were lower than 0.5%.

All the treatments were performed under room temperature (about 18°C±2°C). The pieces of thalli were submerged into seawater during treatment with these inhibitors. The illumination was supplied with white fluorescent lamp (35W) and various levels of light intensities were achieved by adjusting the distance between the thalli and the illumination source. Treated thalli were immediately frozen in liquid nitrogen for later analysis of pigments. A total of three to five independent thallus pieces were treated with each of these inhibitors.

### Pigment extraction and analysis

All pigment extraction procedures were performed under low temperature (0°C) in a dim environment. The preserved thalli were ground in a mortar with liquid nitrogen. Pigments were extracted with 5 ml of methanol: acetone (1∶1, v/v) per 200 mg wet weight of thalli. The extracts were centrifuged for 3 min at 10,000 g, and the supernatants were filtered through a 0.22 µm syringe filter into HPLC vials for HPLC analysis.

Pigment separation and quantification were performed using an Agilent 1200 HPLC equipped with an Rx-C18 analytical column (4.6×250 mm) (Agilent Technologies Inc., Santa Clara, CA, USA). The separation method was modified from Thayer *et al.* [19]. Chlorophyll a, lutein, and Zx standards were obtained from Sigma (St. Louis, MO, USA), and Vx and Ax were obtained from the International Laboratory USA (South San Francisco, CA, USA).

### Measurements of chlorophyll fluorescence

Chlorophyll fluorescence was determined using a Dual-PAM 100 system (Walz GmbH, Effeltrich, Germany). NPQ was calculated as (Fm – Fm′)/Fm′ [20], where Fm represents the maximal chlorophyll fluorescence of the thalli dark adapted for 5 min before any treatment induced by a 300 ms width saturation pulse of 10000 µmol m^−2^s^−1^ and Fm′ represents the maximal chlorophyll fluorescence of the thalli after various treatments.

To evaluate the effects of SA or DCMU on the PSII activity, the thalli of *Ulva* sp. were treated with SA or DCMU for 5 min in the dark before determination of the fluorescence induction curve. The electron transport rate mediated by PSII (ETR (II)) was used to assess the PSII activities. ETR (II) was automatically calculated by the DUAL-PAM software based on the effective PSII quantum yield (Y(II)) of the thalli treated under photosynthetically active radiation (PAR) of 96 µmol m^−2^s^−1^ for about 5 min, i.e. ETR(II) = Y(II)*PAR*0.84*0.5.

### Statistical analysis

All results are presented as mean value ± SD of three to five independent experiments. Statistical analyses were performed using the IBM SPSS Statistics 19 package (IBM Co., Armonk, NY, USA). One-way ANOVA and the Duncan post-hoc test (α = 0.05) were used to test whether statistical differences in the value of the de-epoxidation state (DEPS) existed among various treatment groups. OriginPro 8.5.0 SR1 (OriginLab Co., Northampton, MA, USA) was used to construct graphs and perform linear fitting.

## Results

### Treatment with SA induced accumulation of Ax and Zx in the thalli of *Ulva* sp

**Figure 1:**
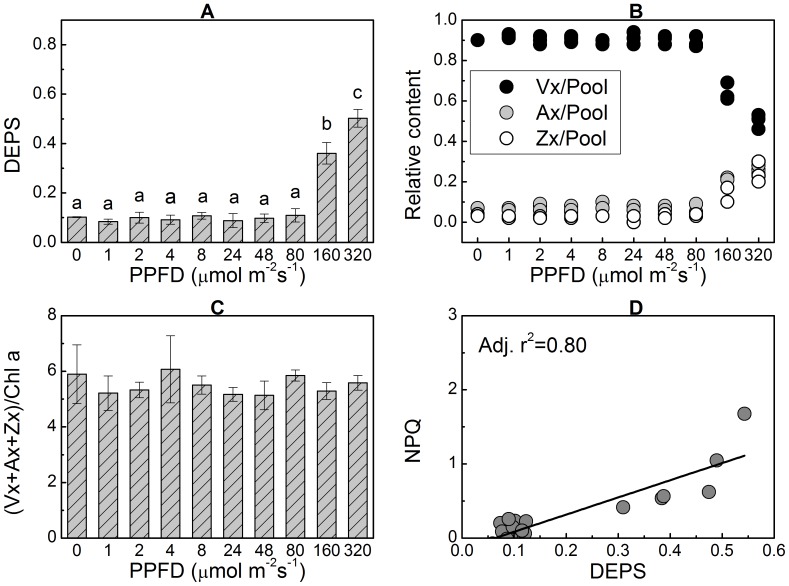
Xanthophyll cycle (Xc) pigment content and consequent NPQ of chlorophyll fluorescence in *Ulva* sp. under various levels of light intensity. The thalli were treated under various levels of light intensity for 1 h. Significant accumulations of Ax and Zx were observed when the PPFD increased to 160µmol m^−2^s^−1^ (A) and were accompanied by a reduction in Vx concentration (B), constant content of total pigments in the Xc pigment pool (C), and increased NPQ of chlorophyll fluorescence (D). The Xc pigment pool size (Vx+Ax+Zx) is presented as µg per mg chlorophyll a, and this also applies to figures 2 and 3. Data are mean value of three independent experiments ± SD. Letters over bars indicate statistical differences based on one-way ANOVA (α = 0.05).

**Figure 2:**
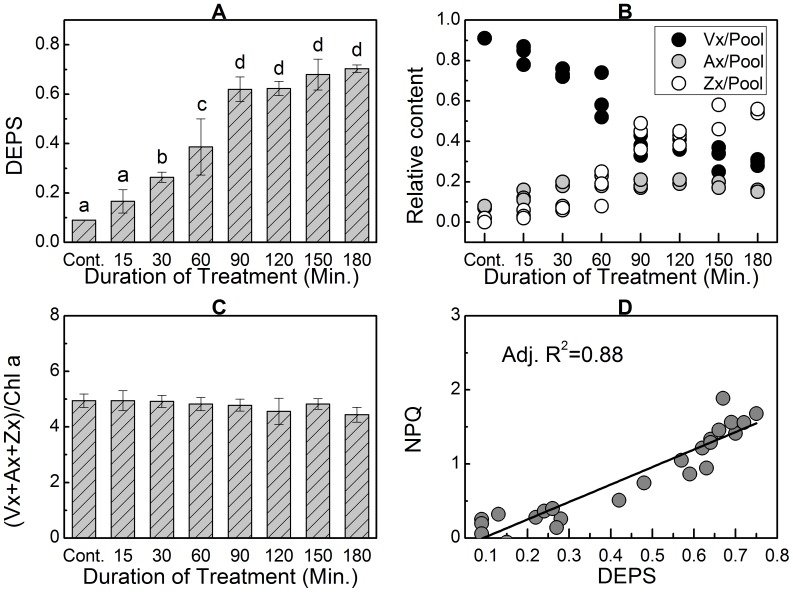
Effects of SA on the xanthophyll cycle (Xc) and NPQ in the thalli of *Ulva* sp. under low light intensity. Significant accumulations of Ax and Zx occurred in the thalli after treatment with SA under very low light condition (8 µmol m^−2^s^−1^) for 30 min or longer (A). This was accompanied by decreased concentration of Vx (B), constant content of total pigments in the Xc pigment pool (C), and increased NPQ (D). Data are mean value of three to five independent experiments ± SD. Letters over bars indicate statistical differences based on one-way ANOVA (α = 0.05).

The DEPS, which was calculated as (Ax+Zx)/(Vx+Ax+Zx), was used to indicate the relative concentrations of Ax and Zx in the Xc pigment pool (i.e., Vx+Ax+Zx) in the thalli of *Ulva* sp. The DEPS in the thalli treated under moderate light conditions (the photosynthetic photon flux density (PPFD) was not higher than 80 µmol m^−2^s^−1^) was about 0.1±0.002 (Fig. 1A). This value increased significantly to 0.36±0.04 when the PPFD increased to 160 µmol m^−2^s^−1^ (P<0.05) and 0.50±0.036 when the PPFD was 320 µmol m^−2^s^−1^ (P<0.05) after 1 h of treatment. The accumulations of Ax and Zx were accompanied by a decrease in the relative content of Vx (Fig. 1B). However, the overall content of the Xc pigment pool remained constant (Fig. 1C), suggesting that these de-epoxidized xanthophylls were converted from Vx. Accumulations of Ax and Zx induced by high light intensity caused nonphotochemical quenching (NPQ) of chlorophyll fluorescence (Fig. 1D). These results suggest that the functional operation of Xc in *Ulva* sp. can be activated by high light illumination (though the tested high light irradiances, i.e. 160 µmol m^−2^s^−1^ and 320 µmol m^−2^s^−1^ might be lower than those *Ulva* sp. often experienced in the field). However, when the thalli were treated with SA, which inhibits ZEP from catalyzing the backward reaction of the Xc [21], significant accumulations of Ax and Zx also occurred, even under very low light intensity (Fig. 2).

Treatment with SA at a final concentration of 5 mM under low PPFD (only 8 µmol m^−2^s^−1^) induced significant accumulations of Ax and Zx in the thalli of *Ulva* sp. (Fig. 2A). The DEPS in the thalli treated with SA for 30 min was 0.26±0.02, which was significantly higher than the control level (0.09±0.001) (P<0.05). This value increased in parallel with the duration of treatment time until 90 min, when DEPS increased to 0.62±0.05. Thereafter, DEPS stabilized after 90 min (P>0.05). SA-induced accumulations of Ax and Zx were also accompanied by a reduction of Vx (Fig. 2B), but the overall content of Xc pigments remained constant (Fig. 2C). This finding suggests that these SA-induced accumulations of Ax and Zx were converted from Vx instead of being newly synthesized from β-carotene. SA-induced accumulations of Ax and Zx under low light illumination led to NPQ; the DEPS and NPQ showed a strong positive linear correlation (the adjusted R^2^ was 0.88) (Fig. 2D).

**Figure 3:**
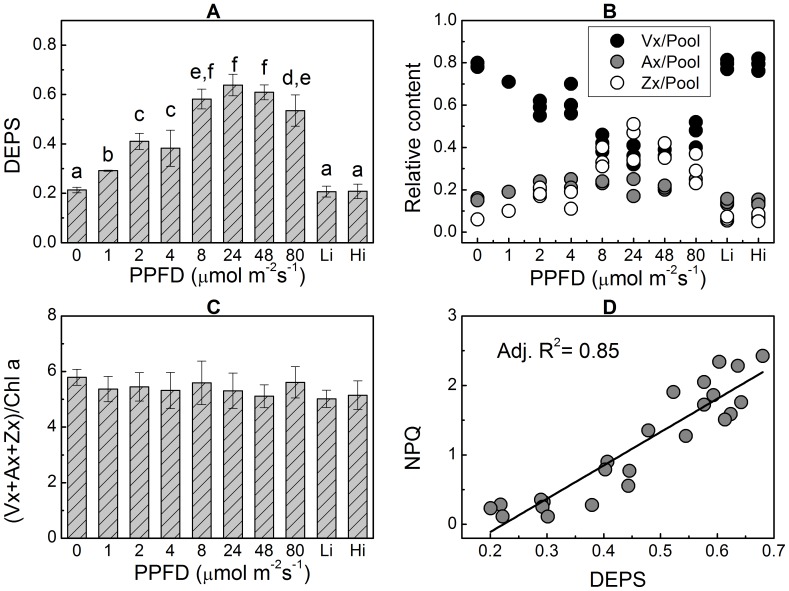
Effects of light intensities on SA-induced accumulations of Ax and Zx in the thalli of *Ulva* sp. Thalli were treated with various light intensities in the presence of SA for 1 h. Within a specific range of PPFD, SA-induced accumulations of Ax and Zx were positively correlated with light intensities (A). When both the PSII- and cytochrome b6f complex-mediated electron transport systems were simultaneously inhibited with DCMU and DBMIB, respectively, the differences in the DEPS in the thalli treated under low light (1 µmol m^−2^s^−1^ (Li)) and relatively high light (24 µmol m^−2^s^−1^ (Hi)) conditions disappeared. Even in the dark (PPFD = 0), SA induced accumulations of Ax and Zx. SA-induced accumulations of Ax and Zx were accompanied by the reduction of Vx concentration (B), constant content of total pigments in the Xc pigment pool (C), and increased NPQ (D). Data are mean value of three to five independent experiments ± SD. Letters over bars indicate statistical differences based on one-way ANOVA (α = 0.05).

To further characterize the phenomenon of SA-induced accumulations of Ax and Zx, we examined the DEPS in the thalli treated with SA under various levels of PPFD for 1 h. Given the side effect of SA on PSII-mediated electron transport (see Figure S1) [13], [22], PPFD was mainly limited within a range of low light to lessen potential photo damage. The results clearly indicate that SA-induced accumulations of Ax and Zx were positively correlated with PPFD within a specific range from darkness to 48 µmol m^−2^s^−1^. While the DEPS in the thalli treated under darkness was 0.213±0.011 (significantly higher than 0.10±0.002 for the thalli without SA (Fig. 1)), the value increased to 0.609±0.030 when the thalli were treated with SA under PPFD of 48 µmol m^−2^s^−1^ (Fig. 3A). However, when the light intensity increased to 80 µmol m^−2^s^−1^, SA-induced DEPS decreased to 0.54±0.06. This may be attributed to light-induced photo damage in the presence of SA, which inhibited about 88.2% of the electron transport within PSII (see Figure S1). SA-induced accumulations of Ax and Zx under various PPFDs were accompanied by decreases of Vx (Fig. 3B), a constant content of the Xc pool (Fig. 3C), and increases of NPQ (Fig. 3D).

Based on the results shown in Fig. 2 and Fig. 3, we concluded that when ZEP was inhibited by SA, the consequent DEPS value depended on two factors: treatment time and light intensity. Thus, when the PPFD was constant, which may imply constant ΔpH across the thylakoid membrane and therefore efficient conversion of Vx to Ax and Zx by VDE, the converted Ax and Zx from Vx would depend on conversion time (Fig. 2). However, when the conversion time was constant (e.g., 1 h in this case), the converted Ax and Zx would depend on the catalytic efficiency. Based on the results shown in Fig. 3, this conversion efficiency is regulated by light intensity. It is well known that higher PPFD is related to a higher ETR mediated by PSII and PSI. Thus, inhibiting PPFD-coupled photosynthetic electron transport would diminish the differences in the DEPS between the thalli treated with SA under very low light (1 µmol m^−2^s^−1^) and those treated under relatively higher light (24 µmol m^−2^s^−1^). When the thalli were treated with SA accompanied by DCMU and DBMIB simultaneously to inhibit PSII- and PSI-mediated electron transport, respectively, the differences between the DEPS in the very low light group and the relatively higher light group were negligible (0.21±0.03 versus 0.22±0.04; P>0.05) (Fig. 3).

### Effects of various inhibitors on SA-induced accumulation of Ax and Zx

**Figure 4:**
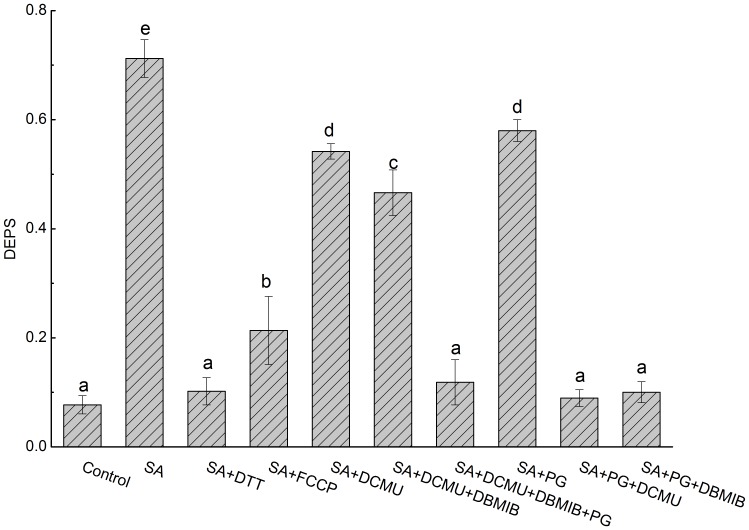
Effects of various metabolic inhibitors on the SA-induced accumulations of Ax and Zx. Thalli were treated with SA and various metabolic inhibitors under low light intensity (8 µmol m^−2^s^−1^) for 1 h. For the final concentrations of inhibitors, see “Materials and Methods.” Data are mean value of three to five independent experiments ± SD. Letters over bars indicate statistical differences based on one-way ANOVA (α = 0.05).

SA-induced accumulations of Ax and Zx were always accompanied by a decreased content of Vx but an invariable content of the Xc pool (Fig. 2 and 3), suggesting that the accumulated Ax and Zx were converted from Vx catalyzed by the enzyme VDE. This is in agreement with the results of the experiment using the VDE inhibitor DTT. When the thalli were treated with DTT and SA simultaneously, SA-induced accumulations of Ax and Zx were significantly inhibited (inhibition percentage was 96.1%±0.039) and the DEPS decreased to 0.102±0.025, which was comparable to that of the control group (0.078±0.017) (P>0.05) (Fig. 4).

Activation of VDE reportedly is regulated by ΔpH, in that a high concentration of protons stimulates VDE activities [23]. This is consistent with the results of the FCCP experiment (Fig. 4). FCCP collapses the proton gradient across the thylakoid membrane [15], and its presence significantly diminished the SA-induced accumulations of Ax and Zx. Its inhibition percentage was 78.6%±0.098, and the DEPS decreased from 0.712±0.035 to 0.213±0.025 (P<0.05). This result suggests that the proton gradient across the thylakoid membrane activates VDE, which in turn catalyzes the conversion of Vx to Ax and Zx.

In the chloroplast, the proton gradient across the thylakoid membrane is always coupled with electron transport. Thus, inhibiting electron transport systems should eliminate SA-induced accumulations of Ax and Zx. As is shown in Fig. 4, in the presence of SA, adding DCMU to completely block electron transport mediated by PSII resulted in a significant decrease of the DEPS (the inhibition percentage was 26.8%±0.022). The DEPS was decreased further when SA, DCMU, and DBMIB were added simultaneously (the inhibition percentage was 38.7%±0.066), which suggests that both PSII-mediated linear electron transport (LEF) and PSI-mediated cyclic electron transport (CEFI) systems participated in the activation of VDE. To our surprise, without photosynthetic electron transport (i.e., in the dark, PPFD was zero in Fig. 3A) or when photosynthetic electron transport systems were inhibited by DCMU and DBMIB simultaneously under light illumination (Li and Hi group in Fig. 3A, Fig. 4), SA still induced significant accumulations of Ax and Zx. This finding implies that other alternative electron transport pathway exists. The most likely pathway is chlororespiration, in which plastoquinones are reduced by the NAD(P)H dehydrogenase complex (Ndh) and then oxidized by PTOX [24], [25]. Thus, when PTOX was inhibited by PG and photosynthetic electron transport systems, including LEF and CEFI, were inhibited by DCMU and DBMIB, the DEPS in the SA-treated thalli decreased to 0.118±0.041, which was very close to the control level (0.077±0.017) (P>0.05) (Fig. 4).

It is noteworthy that the effects of PG on the SA-induced accumulations of Ax and Zx were complicated (Fig. 4). When only PG was applied with SA, its inhibition effect was limited (inhibition percentage was about 20.8%±0.032), causing the DEPS to decrease from 0.712±0.035 to 0.58±0.02 (P<0.05). However, when PG and DCMU or PG and DBMIB were applied simultaneously with SA, the DEPS decreased to 0.089±0.015 and 0.1±0.019, respectively (the inhibition percentage was 98.1%±0.024 and 96.4%±0.03, respectively), and both were identical to the control level (both P>0.05).

## Discussion

### SA inhibits not only ZEP but also most of the PSII activities in *Ulva* sp

The results above were obtained based on the inhibition of ZEP by SA, so the influence of SA on other physiological process should be discussed. The inhibition effect of SA on ZEP was first reported by Hager (1966) [21], and afterwards it was utilized in the investigations of Xc in rice [12] and marine macro-algae [26]. However, Berg and Izawa (1976) reported that SA influences the photosynthetic electron transfer systems in many ways [13]. SA inhibits both PSII- and PSI-mediated electron transport at a high final concentration (approximately 45 mM), whereas at low concentration (less than 10 mM) SA affects only electron transport mediated by PSII (its effect on PSI is negligible) [13], [22], [27], [28]. Therefore, we selected a working concentration of SA of 5 mM to inhibit ZEP [12] and concurrently to minimize its side effects on the other metabolic pathways. The Figure S1 shows that about 88.2% of PSII activity in *Ulva* sp. was suppressed by SA at 5 mM. Thus, about 11.8% of activity of PSII remained. This result is in agreement with the inhibition effect of SA on the oxygen evolution in the chloroplast [13]. It is therefore that the impact on VDE activity of ΔpH coupled with the alternative electron transports other than linear electron transport was further discussed.

### Under low light conditions both VDE and ZEP are active to operate the Xc

There was no significant accumulation of Ax and Zx in the thalli of *Ulva* sp. when the PPFD was less than 160 µmol m^−2^s^−1^ (Fig. 1). However, when the backward reaction (from Zx to Vx) catalyzed by ZEP was inhibited by SA, large amounts of Ax and Zx were accumulated at the expenses of Vx under low light intensity (<160 µmol m^−2^s^−1^) (Fig. 2 and Fig. 3), suggesting that the low level of DEPS under low PPFD (Fig. 1) results from the catalytic activity of ZEP rather than from the inactivation of VDE. In other words, both the forward (from Vx to Zx) and the backward (from Zx to Vx) reactions of the Xc operate under low light conditions, and accumulations of Ax and Zx depend on the different activities of VDE and ZEP. As a consequence, one explanation for the accumulations of Ax and Zx induced by high light intensity (Fig. 1) could be that the VDE activity significantly increased and had higher activity than ZEP. On the other hand, suppressing or weakening the activity of ZEP also could induce significant accumulations of Ax and Zx in the absence of high light intensity. Therefore, any factors that either up-regulate VDE activity or down-regulate ZEP activity may lead to significant accumulations of Ax and Zx, which is clearly demonstrated in Fig. 2 and 3. Takahashi *et al.* (2006) reported that mutation of the chloroplast NAD kinase 2 in *Arabidopsis thaliana* caused sustained high concentration of Ax and Zx, which the authors ascribed to impaired ZEP activity caused by deficiency of cofactor NADPH due to the mutation of *nadk2* [29].

We propose that the Xc in *Ulva* sp. inhabiting the intertidal zone can be divided into two types in terms of accumulations of Ax and Zx. The first type is characterized by the reversible accumulation of Ax and Zx induced by high light conditions, and the second type is characterized by both activity of VDE and ZEP under moderate conditions but without accumulation of Ax and Zx. These two types of Xc could be operating under different environmental conditions. Apparently, the accumulation of Ax and Zx can function in NPQ, but the function of the second type of cycle is less apparent. Under moderate light conditions, the Xc pigments would keep dynamic between lipid and LHCII subunits because of the permanent cycling of VDE and ZEP. Thus, there would always be certain amounts of xanthophyll molecules retained within the lipid phase, especially Zx, which could regulate the fluidity of the thylakoid membrane [30] and also protect the thylakoid membrane from oxidative damage [31], [32], [33]. In addition, the epoxidation of this transient Zx would reduce potential production of reactive oxygen species (ROS) by consuming oxygen, which is introduced into Zx by ZEP [34], [35]. Overall, two molecules of dioxygen and four molecules of NADPH (two for the regeneration of ascorbate consumed by VDE in de-epoxidation and another two for the epoxidation catalyzed by ZEP) would be consumed, producing four molecules of water to finish a cycle(one molecule of Vx de-epoxidized to Zx and then back to Vx). For intertidal macro-algae, which frequently experience desiccation and consequent oxidative damage, regulation of membrane fluidity and reduction of the dioxygen level would be very important for survival.

### Positive relationship between VDE activity and alternative electron transports

The activity of VDE requires ΔpH across the thylakoid membrane [36] (Fig. 4). How this ΔpH is generated under low light or dark conditions was investigated in this study. The ΔpH across the thylakoid membrane required for the activity of VDE is built by coupling with electron transport systems, which involve multiple pathways. The results of the inhibitor study with DCMU, which completely inhibits PSII-mediated electron transport, suggest that PSII-mediated LEF was not compulsory for the activation of VDE (Fig. 4). Thus, other alternative electron transport pathways must have contributed to the activation of VDE. The results obtained from the DCMU and DBMIB study support the premise that CEFs also participated in the activation of VDE. Besides, as shown in Fig. 3, chlororespiration-coupled proton pumping could activate VDE. SA could induce accumulations of Ax and Zx in the dark (the zero group in Fig. 3) or under light illumination when the PSII- and PSI-mediated electron transport systems were completely inhibited with DCMU and DBMIB, respectively (the Li group and Hi group in Fig. 3). The PG inhibitor study confirmed the participation of chlororespiration in the activation of VDE (Fig. 4).

In higher plants, the ETRs mediated by alternative pathways are very low. For example, CEFI corresponds to only ∼30% of LEF during photosynthetic induction in wild-type potato (i.e., the ratio between electron flux through PSI and PSII was usually around 1.3) [37], while chlororespiration corresponds to only 3% of LEF [38]. Thus, it is reasonable to assume that the ΔpH required for the activation of VDE in *Ulva* sp. is relatively very low, which should be the prerequisite for the parallel operation of VDE and ZEP in *Ulva* sp.

### Chlororespiration functions in building up the ΔpH and diminishing the dioxygen level in chloroplasts

The results shown in Fig. 4 suggest that the inhibition effects of PG on the VDE activity are complicated. When PG was applied with DCMU and/or DBMIB simultaneously in the presence of SA, its inhibition effects were significantly higher than when only PG was applied with SA. Furthermore, when PG was used with DCMU and SA simultaneously to inhibit chlororespiration, LEF, and ZEP, respectively, thus leaving CEFI to operate, CEFI did not activate VDE (Fig. 4). This unexpected result seems to contradict the results obtained from the inhibitor study using DCMU and DBMIB in the presence of SA (Fig. 4). This apparent contradiction may be explained by the multiple effects of PTOX in the chloroplast.

**Figure 5:**
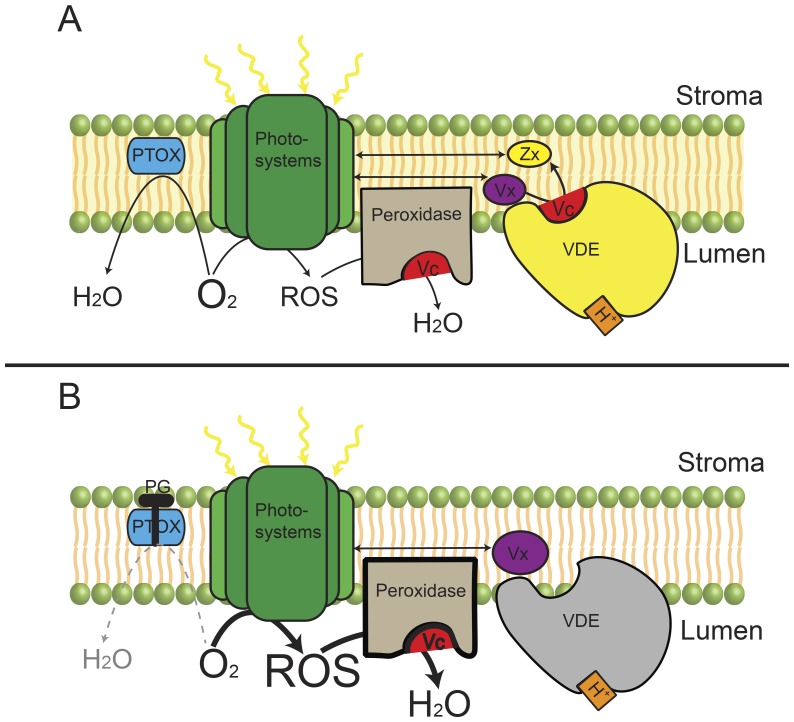
Presumed paradigm of how PTOX indirectly influenced VDE activity in the presence of DCMU and SA. DCMU inhibited PSII activity completely, thus inevitably leading to the formation of ROS via the interaction of oxygen and excited chlorophylls in PSII. PTOX-involved electron transport consumed oxygen and therefore partly reduced the formation of ROS (A). However, when PG was applied simultaneously with SA and DCMU (B), large amounts of ROS would form, which drained the supply of reduced ascorbate. Because regeneration of reduced ascorbate was severely retarded in the presence of DCMU, inhibition of PTOX with PG in the presence of DCMU and SA not only influenced the build-up of ΔpH but also indirectly affected the VDE activity by snatching reduced ascorbate. Dashed lines represent the blocked reactions. For the clarity of draw, DCMU and SA are not shown in both A and B. PTOX: plastid terminal oxidase; ROS: reactive oxygen species; VDE: violaxanthin de-epoxidase; Vx: violaxanthin; Zx: zeaxanthin.

In higher plants and green algae, PTOX is thought to be involved in chlororespiration by mediating the oxidization of plastoquinol and the reduction of dioxygen to form water [25], [39]. This process could be electrogenic when coupled with type I-Ndh or PSII [40], [41], but it also could diminish the dioxygen level around the thylakoid membrane [42], [43], [44]. Thus, inhibition of PTOX with PG would result not only in cessation of PTOX-mediated electron transport but also in the disappearance of the PTOX-created relatively low oxygen environment (Fig. 5). Molecular oxygen is potentially harmful, especially when the electron flow is blocked by DCMU, which blocks electron transfer from Q_A_ to Q_B_. In this case, excited PSII reaction centers cannot transfer their electrons to the next acceptors, causing the formation of triplet chlorophylls [2], [45]. These chlorophylls could interact with dioxygen to form singlet state oxygen or superoxide radicals (i.e., ROS) [42], [46], [47]. Therefore, when PG, SA, and DCMU were applied simultaneously, large amounts of ROS would inevitably be produced (Fig. 5B). It is noteworthy that in the chloroplast, scavenging of ROS primarily depends on the reduced ascorbate through ascorbate peroxidase [42], [47], [48]. When the main system for photosynthetic electron transport along the thylakoid membrane has been blocked by DCMU, regeneration of ascorbate from dehydroascorbate is severely retarded due to the lack of a reducing equivalent [48]. Thus, increased production of ROS would drain most of the reduced ascorbate in the chloroplast. Given that reduced ascorbate is also required for VDE to convert Vx to Ax and Zx and that deficiency of reduced ascorbate would limit VDE activity [49], infiltration of PG to inhibit PTOX in the presence of DCMU and SA would also restrict the VDE activity by snatching away the reduced ascorbate. Thus, although a proton gradient coupled with CEF may exist in the presence of SA, DCMU, and PG, significant accumulations of Ax and Zx did not occur.

## Supporting Information

Figure S1 **Effects of SA and DCMU on the PSII activity in** ***Ulva*** **sp.** The thalli were treated with SA at 5 mM or DCMU at 10 µM for 5 min in the dark before the fluorescence determination. The photosynthetically active radiation was set as 96 µmol m^−2^s^−1^. Data are mean value of three independent experiments ± SD.
(TIF)
