# Correction: Alternative Electron Transports Participate in the Maintenance of Violaxanthin De-Epoxidase Activity of *Ulva* sp. under Low Irradiance

**DOI:** 10.1371/annotation/851cec52-b3d4-40bb-a857-e59b1a7a6c88

**Published:** 2014-01-03

**Authors:** Xiujun Xie, Wenhui Gu, Shan Gao, Shan Lu, Jian Li, Guanghua Pan, Guangce Wang, Songdong Shen

The grants from the National Natural Science Foundation of China listed in the Funding Statement are incorrect. The correct grant numbers are: 41176137, 41176134 and 41106131. 

